# Extensive metabolic consequences of human glycosyltransferase gene knockouts in prostate cancer

**DOI:** 10.1038/s41416-022-02040-w

**Published:** 2022-11-08

**Authors:** Michèle Rouleau, Flora Nguyen Van Long, Véronique Turcotte, Patrick Caron, Louis Lacombe, Armen Aprikian, Fred Saad, Michel Carmel, Simone Chevalier, Eric Lévesque, Chantal Guillemette

**Affiliations:** 1 Centre Hospitalier Universitaire de Québec Research Center - Université Laval (CRCHUQc-UL), Faculty of Pharmacy and Centre de Recherche sur le Cancer, Université Laval, Québec, Canada; 2 CRCHUQc-UL and Faculty of Medicine—Université Laval, Québec, Canada; 3 McGill University Health Centre, Faculty of Medicine, McGill University, Québec, Canada; 4 Centre Hospitalier de l’Université de Montréal, Faculty of Medicine, Université de Montréal, Québec, Canada; 5 Faculty of Medicine, Université de Sherbrooke, Québec, Canada

**Keywords:** Biomarkers, Cancer

## Abstract

**Background:**

Naturally occurring germline gene deletions (KO) represent a unique setting to interrogate gene functions. Complete deletions and differential expression of the human glycosyltransferase *UGT2B17* and *UGT2B28* genes are linked to prostate cancer (PCa) risk and progression, leukaemia, autoimmune and other diseases.

**Methods:**

The systemic metabolic consequences of UGT deficiencies were examined using untargeted and targeted mass spectrometry-based metabolomics profiling of carefully matched, treatment-naive PCa cases.

**Results:**

Each UGT KO differentially affected over 5% of the 1545 measured metabolites, with divergent metabolic perturbations influencing the same pathways. Several of the perturbed metabolites are known to promote PCa growth, invasion and metastasis, including steroids, ceramides and kynurenine. In *UGT2B17* KO, reduced levels of inactive steroid-glucuronides were compensated by sulfated derivatives that constitute circulating steroid reservoirs. *UGT2B28* KO presented remarkably lower levels of oxylipins paralleled by reduced inflammatory mediators, but higher ceramides unveiled as substrates of the enzyme in PCa cells.

**Conclusion:**

The distinctive and broad metabolic rewiring caused by UGT KO reinforces the need to examine their unique and divergent functions in PCa biology.

## Graphical abstract

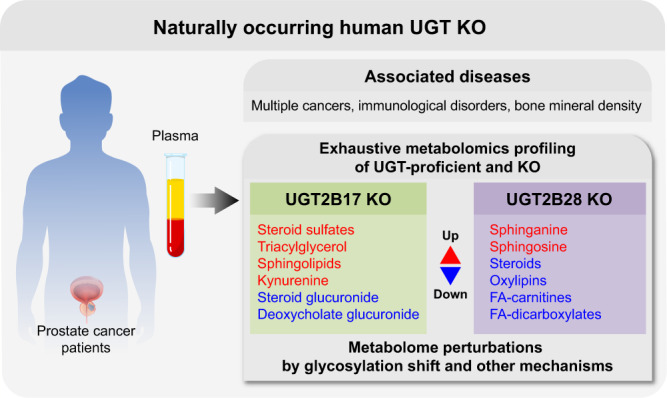

## Background

**Fig. 1:**
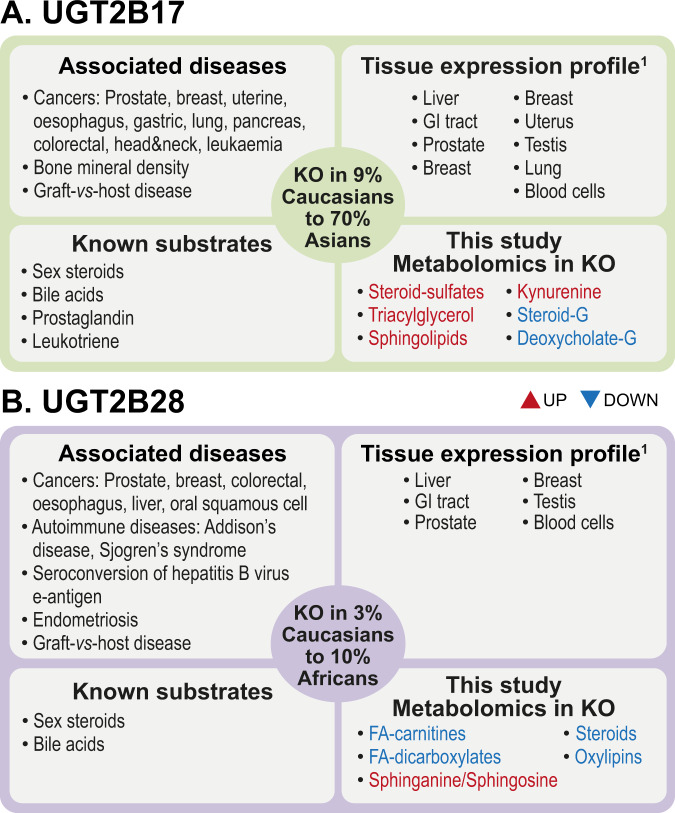
Summary of current clinical and biochemical knowledge on human UGT2B17 and UGT2B28. Frequencies of UGT complete gene deletions (KO) vary among ethnic groups. (a) UGT2B17 homozygous gene deletion: Caucasians: 9%; Africans: 4%; Asians: 70% [7]. (b) UGT2B28 homozygous gene deletion: Caucasians: 3%; Africans: 10%; Asians: 2% (based on the frequency of the tag SNP rs12507041 GG genotype reported in the 1000 Genome project Phase 3 (https://m.ensembl.org/Homo_sapiens/Variation/Population?db=core;r=4:69395647-69396647;v=rs12507041;vdb=variation;vf=96007771; searched May 31, 2021). References to relevant literature are provided in the text. FA fatty acids, G UGT-derived glucuronide products, GI gastrointestinal. ^1^Complete tissue expression profile is provided in Supplementary Fig. S1.

Defining the global function of human genes is challenging and often relies on studying genes of interest in other vertebrates or cell models amenable to genetic manipulations. Naturally occurring homozygous loss-of-function variants and gene deletions (KO) in humans represent unique settings to interrogate global gene functions and relationships with clinical conditions [1]. For example, loss-of-function variants of the *APOC3* and *PCSK9* genes reduce levels of triglycerides and low-density lipoprotein, respectively, and afford protection against coronary heart disease [2, 3]. Such cases are rare, and variants that inactivate genes are often missed by studies relying on exome array sequencing [4]. The glycosyltransferase enzymes UGT2B17 and UGT2B28 are encoded by two of the ten most commonly deleted genes of the human genome [5]. By contrast, no complete deletion of other UGTs, including the highly related *UGT2B15* gene, has been experimentally documented [6]. Frequencies of complete homozygous *UGT2B17* and *UGT2B28* gene deletions vary from 4% in Africans to 9% in Caucasians and 70% in Asians for *UGT2B17* and from 2% in Asians and 3% in Caucasians to 10% in Africans for *UGT2B28* (Fig. 1) [7, 8]. *UGT2B17* and *UGT2B28* germline deletions and differential expression have been associated with a number of clinical conditions such as risk and progression of prostate cancer (PCa), but also leukaemia, oesophageal, colorectal, lung, bladder and breast cancers [9–14]. They have been also linked to other conditions such as bone mineral density and osteoporosis, graft-versus-host diseases, endometriosis as well as Sjogren’s syndrome and Addison’s disease, two autoimmune diseases of salivary/lacrimal and adrenal glands, respectively (Fig. 1) [15–23]. However, the mechanisms underlying the links between deficiencies in UGT2B17 and UGT2B28 metabolic pathways and numerous diseases such as PCa remain largely unknown.

UGT2B17 and UGT2B28 are part of a family of 22 glycosyltransferases best known for their detoxification functions targeting drugs and other xenobiotics, mainly exerted in the liver, gastrointestinal tract and kidneys. They also participate in the homoeostasis of endogenous molecules, such as steroid hormones, in a variety of tissues, including the prostatic tissue, but their endogenous functions remain largely unexplored, and especially for UGT2B28 (Fig. 1 and Supplementary Fig. S1) [24]. In fact, the enzymatic function of the UGT2B28 protein has been studied in only one previous study, based on its overexpression in HEK293 cells [25]. Based on this limited understanding, one underlying mechanism for their contribution to the aetiology and aggressiveness of some diseases relates to a perturbed steroidome (steroid metabolome), evidenced in biological fluids and tissues in diverse clinical settings, including hormone-sensitive conditions such as PCa, puberty and doping [10, 13, 20, 26–31]. A few previous ‘omics studies have also identified perturbations of the UGT-associated “pentose and glucuronate interconversion pathway” in a core metabolic signature associated with several cancers, including advanced PCa [32–34].

Building on these observations and given their wide tissue distribution (Supplementary Fig. S1), we hypothesised that UGT2B17 and UGT2B28 deficiencies induce a significant rewiring of the systemic metabolome to which cancer cells are exposed, driven by several tissues. A broader knowledge of UGT metabolic influence at the systemic level could help explain the aetiology of associated clinical conditions and identify possible novel therapeutic targets. To this end, we comprehensively profiled and compared the circulating metabolome of PCa individuals with complete loss of the *UGT2B17* or *UGT2B28* genes and gene-proficient individuals. By untargeted and targeted mass spectrometry (MS) approaches, we identify an unexpected global rewiring of metabolism in both classes of KO individuals well beyond steroid hormones. Still, we reveal divergent metabolic perturbations in each KO group and different classes of metabolites affected by the absence of functional UGT2B17 and UGT2B28 pathways. We thus expose several important metabolic perturbations in KO PCa cases that help to understand how UGT2B17 and UGT2B28 influence PCa progression.

## Methods

### Study settings/cohort

Patients from the PROCURE Prostate Cancer cohort were recruited between 2007 and 2012 at four university hospital centres in the Province of Québec, Canada (Montréal, McGill, Québec and Sherbrooke) [35]. The cohort included over 2032 male patients with localised PCa at the time of diagnosis, of which 2007 underwent radical prostatectomy. The study was performed in accordance with the Declaration of Helsinki. Before surgery, each patient provided written informed consent for research and the protocol was evaluated and approved local Ethical Research Committee (CHUQc-UL #2012-362). Patients were screened as described previously to determine the germline *UGT2B17* and *UGT2B28* genetic status [36]. Deletion frequencies were in accordance with those of Caucasian populations [7, 8]. The metabolomics analysis was conducted on 84 plasma samples collected before prostatectomy from treatment-naive patients, to avoid potential interference by hormonal treatment, known to regulate UGT gene expression [37]. Patients had a complete germline deletion of both copies of the *UGT2B17* (*n* = 30; no deletion of *UGT2B28*) or both copies of *UGT2B28* (*n* = 24; no deletion of *UGT2B17*) genes, or were *UGT2B17/UGT2B28*-gene-proficient controls (*n* = 30; i.e., two functional copies of each gene). These patients were matched for age and adverse pathological and clinical features (prostate serum antigen (PSA), Gleason score and tumour stage). Characteristics of matched groups are provided in Table 1.

**Table 1:** Clinical and pathological characteristics of matched samples in UGT groups.

Characteristics	Controls^a^	*UGT2B17* KO^b^	*UGT2B28* KO^c^
	*n* = 30	*n* = 30	*n* = 24
Mean age at diagnosis ± SD (years)	61.7 ± 5.6	64.0 ± 4.9	62.6 ± 5.6
Range	51.7–70.7	53.8–73.4	53.7–73.7
PSA at diagnosis ± SD (ng/ml)	7.4 ± 7.5	7.0 ± 5.0	7.6 ± 4.7
Range	2.3–45.4	1.2–21.0	3.9–24.2
	***n*** **(%)**	***n*** **(%)**	***n*** **(%)**
Pathologic Gleason score			
<7	6 (20%)	7 (23%)	5 (21%)
7	23 (77%)	22 (74%)	18 (75%)
>7	1 (3%)	1 (3%)	1 (4%)
Pathologic T stage			
<pT3a	15 (50%)	18 (60%)	13 (54%)
pT3a	13 (43%)	10 (33%)	9 (38%)
>pT3a	2 (7%)	2 (7%)	2 (8%)

^a^Subjects carrying both copies of the *UGT2B17* and *UGT2B28* genes.

^b^Subjects homozygous for *UGT2B17* gene deletion (KO) and carrying both copies of the UGT2B28 gene

^c^Subjects carrying both copies of the *UGT2B17* gene and homozygous for *UGT2B28* gene deletion (KO).

PSA: prostate serum antigen at diagnosis; age at PCa diagnosis.

### Metabolomics quantification methods

#### Untargeted global metabolomics

Plasma sample aliquots were analysed for profiling of global metabolites by the metabolomics platform at Metabolon Inc. (Durham, NC, USA). Samples were prepared using the automated MicroLab STAR system (Hamilton Company, Reno, NV, USA). A recovery standard was added prior to the first step in the extraction process for quality control purposes. Metabolites were extracted by vigorous agitation after the precipitation of proteins with methanol. The resulting extract was divided in four fractions analysed as follows: two for reverse phase (RP)/ultra-performance liquid chromatography (UPLC)-MS/MS methods with positive ion mode electrospray ionisation (ESI), one for analysis by RP/UPLC-MS/MS with negative ion mode ESI, one for analysis by HILIC/UPLC-MS/MS with negative ion mode ESI utilising a Waters ACQUITY UPLC system coupled to a Thermo Scientific Q-Exactive high-resolution MS equipped with a heated ESI source and an Orbitrap mass analyser. Raw data extraction, peak identification, and quality control processing were carried out using the Metabolon proprietary hardware and software. Compound identification was done through comparison with a library of chromatographic and MS data from authenticated standards. Peaks were quantified using the area under the curve (AUC) method, and data were normalised for inter-day signal differences. The analytical variability was ≤10%.

#### Lipidomics

Complex lipid profiling was conducted according to a modified version of a previously described protocol by the metabolomics platform at Metabolon Inc [38]. Lipids were extracted from plasma samples by a heptane/ethyl acetate mixture after the addition of a butanol/methanol solution. Phase separation was induced by the addition of aqueous acetic acid and centrifugation. MS analysis was conducted on a Shimadzu LC with nano PEEK tubing coupled to a Sciex SelexIon-5500 QTRAP. The scan was performed in multiple reaction monitoring mode. Peaks were quantified using the AUC method, and data were normalised for inter-day signal differences. Individual lipid species were quantified by taking the peak area ratios of target compounds and their assigned internal standards, then multiplying by the concentration of internal standard added to the sample. The analytical variability was ≤10%.

#### Steroid quantification

Plasma steroid levels were measured by LC/MS-MS and GC-MS using previously published methods [39, 40]. In the first assay, ten unconjugated classical steroids were measured using 250 µL of plasma, whereas two sulfated and three glucuronidated classical steroids were measured in two independent assays using 20 µL and 100 µL of plasma, respectively. For the second assay, seven 11-oxygenated C19 androgens were measured with 200 µL of plasma. Analyses were performed in a blinded fashion. Reference steroids were purchased from Steraloids (Newport, RI, USA), Cambridge Isotope (Tewksbury, MA, USA) and Iso-Science (Ambler, PA, USA). Internal deuterated steroid standards were added to samples, and quality controls were included in each run. The measured steroids and their limits of quantification were as follows: steroids in assay #1: dihydroepiandrosterone (DHEA), 100 pg/mL; progesterone, 50 pg/mL; androstenediol, 50 pg/mL; testosterone, 30 pg/mL; DHT, 10 pg/mL; androsterone, 50 pg/mL; androstane-3β, 17β-diol, 10 pg/mL; estrone, 5 pg/mL; estradiol, 1 pg/mL; androstenedione, 50 pg/mL; androsterone-glucuronide, 1 ng/mL; androstane-3α,17β-diol-3-glucuronide, 0.25 ng/mL; androstane-3α,17β-diol-17-glucuronide, 0.25 ng/mL; DHEA-sulfate, 0.075 mg/mL; estrone-sulfate, 0.075 ng/mL. Steroids measured in assay #2 were the adrenal-derived 11-hydroxyandrostenedione, 11-keto-androstenedione; the androgenic 11-keto-testosterone, 11-keto-dihydrotestosterone, 11-hydroxytestosterone, and their metabolites 11-hydroxyandrosterone and 11-keto-androsterone, each with a lower limit of quantification of 10 pg/ml. Three low and three high-hormone concentration quality control replicates were included in each run, and all metabolite coefficients of variation were <10%. Steroid measures in urine samples were from a previous study [41].

#### Oxylipin quantification

Oxylipins were quantified in plasma samples at the West Coast Metabolomics Center (University of California at Davis, CA, USA) as previously described [42]. Internal standards were added to plasma samples prior to extraction with acetonitrile/methanol (50:50). MS analysis was conducted by MS/MS on an API 4000 QTrap (Sciex, Framingham, MA) in negative mode ESI.

### In vitro enzymatic assays

Glycosyltransferase activity was assessed with protein preparations from human livers as a positive control (HLM, Xenotech, Lexena, KS, USA) and LNCaP and LAPC4 PCa cell models expressing UGT2B17 or UGT2B28, as indicated in the legend of figures. Reaction assays contained 50 µg homogenates or 20 µg microsomal proteins, 50 mM Tris-HCl (pH 7.5), 10 mM MgCl_2_, 5 μg/mL pepstatin, 0.5 μg/mL leupeptin, 0.5 mM UDP-glucuronic acid, 20 μg/mL alamethicin and 200 μM of substrates in a final volume of 100 μL. Reaction assays were incubated at 37 °C for 4 hours, stopped with 100 μL methanol. Chemicals were from Sigma (St. Louis, MO, USA). Glucuronide formation was assessed by LC/MS-MS as previously described [39, 43].

### Data analysis and statistics

Samples included 24 matched triplets of *UGT2B28* KO, *UGT2B17* KO and gene-proficient controls and six additional pairs of *UGT2B17* KO and gene-proficient cases. Metabolomics data were log-transformed prior to statistical comparisons using paired sample two-sided *t* test, and fold changes (FCs) were calculated based on the mean. The 30 *UGT2B17* KO were compared with the 30 matched gene-proficient controls. The 24 *UGT2B28* KO samples were compared with the 24 matched gene-proficient controls and with all 30 controls, with similar conclusions. Compiled metabolomics data and statistical analysis are provided in Supplementary Tables S1, S2, S3A-B and S4A–B. Complete metabolomics quantitative data per patient are provided in Supplementary Table S5. Pathway enrichment analyses using global untargeted metabolomics data were performed with Metabolon online tools and using their proprietary database. The enrichment score was calculated by dividing the ratio of statistically significantly changed metabolites within a pathway by the overall proportion of statistically significantly changed metabolites.

### Reporting summary

Further information on research design is available in the Nature Research Reporting Summary linked to this article.

## Results

### Overview of the comprehensive profiling of the circulating metabolome

**Fig. 2:**
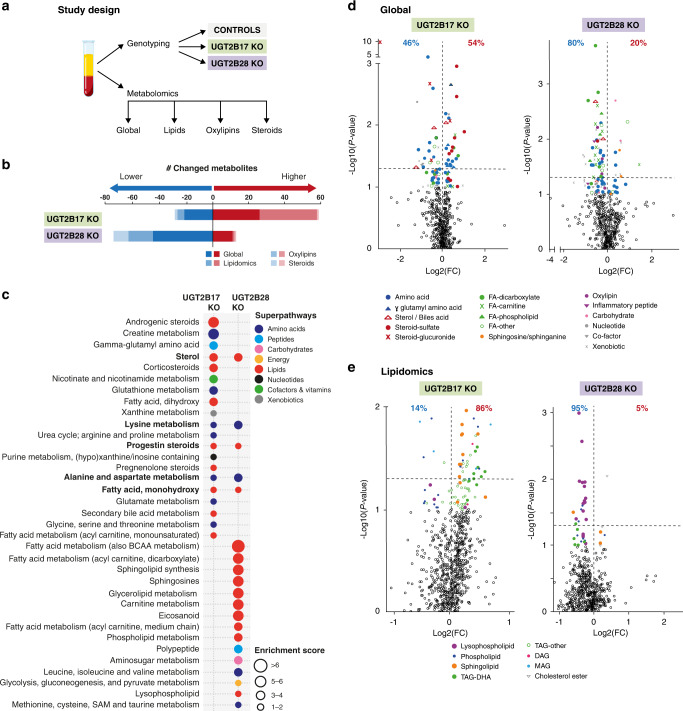
Summary of circulating metabolites changed in KO vs gene-proficient controls. **a** Experimental design. **b** Number of changed metabolites in KO cases (higher than controls: red; lower than controls: blue; *P* ≤ 0.05). **c** Main global metabolic pathways perturbed in KO cases. Superpathways and Enrichment scores are according to Metabolon, as described in 'Methods'. **d**, **e** Volcano plots of **d** global and **e** lipidomics data. % metabolites higher or lower than control individuals are given (detailed in Supplementary Table S1). The statistical significance of fold change (FC) was determined by paired test on log-transformed data. DAG diacylglycerol, DHA docosahexanoic acid, FA fatty acids, KO knock out, MAG monoacylglycerol, TAG triacylglycerol.

Men with localised PCa from the PROCURE cohort were carefully matched for age and prognostic characteristics (Table 1). To increase the metabolome coverage, we used three platforms for untargeted and targeted metabolomics for characterisation of preoperative plasma specimens from 30 *UGT2B17* KO, 24 *UGT2B28* KO and 30 gene-proficient treatment-naive PCa cases undergoing prostatectomy (Fig. 2a). Among the 1545 measured metabolites, 89 (5.8%) and 88 (5.7%) metabolites were significantly changed in *UGT2B17* KO and *UGT2B28* KO respectively, relative to control individuals (Supplementary Table S1). In *UGT2B17* KO, a majority of changed metabolites were higher than in gene-proficient cases (66% of changed metabolites were increased). By contrast, a large majority of changed metabolites in each class were lower in *UGT2B28* KO individuals relative to gene-proficient cases (85% of changed metabolites were decreased) (Fig. 2b and Supplementary Table S1).

The untargeted global metabolomics assay readily pinpointed lipids as the main perturbed superpathway in both sets of KO cases, especially for cholesterol-derived steroid hormones and bile acids, and fatty acid-related metabolites (Fig. 2c). However, each KO was mainly characterised by distinctive metabolic changes. For *UGT2B17* KO, five of the ten most upregulated metabolites were steroid sulfate conjugates (fold change (FC) of 1.4–2.1X; *P* < 0.05), whereas two of the most downregulated metabolites were glucuronide conjugates, with deoxycholate-glucuronide (FC −8.3X, *P* < 10^–6^) being the most discriminating metabolite in *UGT2B17* KO individuals (Fig. 2d and Supplementary Table S2). In *UGT2B28* KO individuals, in addition to cholesterol-derived metabolites, several subclasses of fatty acids and especially carnitines and dicarboxylates as well as eicosanoids, were the most perturbed (Fig. 2c). Among the very few elevated metabolites in *UGT2B28* KO were the sphingolipid precursors sphinganine and sphingosine (FC 1.5–1.6X; *P* < 0.05) whereas two markers of inflammation, leukotriene B4 and the peptide HWESASLLR, reduced by 12% and 33%, respectively, were among the most discriminating metabolites of *UGT2B28* KO (Fig. 2d and Supplementary Table S2). Several amino acid-related pathways were also affected in both KO groups (Fig. 2c, d and Supplementary Table S3A). The lipidomics analysis further identified several divergent metabolic perturbations between UGT KO groups and especially the fact that 86% of perturbed circulating lipid species were significantly higher in *UGT2B17* KO, whereas in *UGT2B28* KO cases 95% perturbed metabolites were significantly lower, compared to controls (Fig. 2e and Supplementary Table S3B). Clearly, untargeted metabolomics and lipidomics assays highlighted the significantly perturbed yet unique circulating metabolome between *UGT2B17* and *UGT2B28* KO cases.

### Distinctive impact of *UGT2B17* KO and *UGT2B28* KO on the circulating steroidome and bile acids

**Fig. 3:**
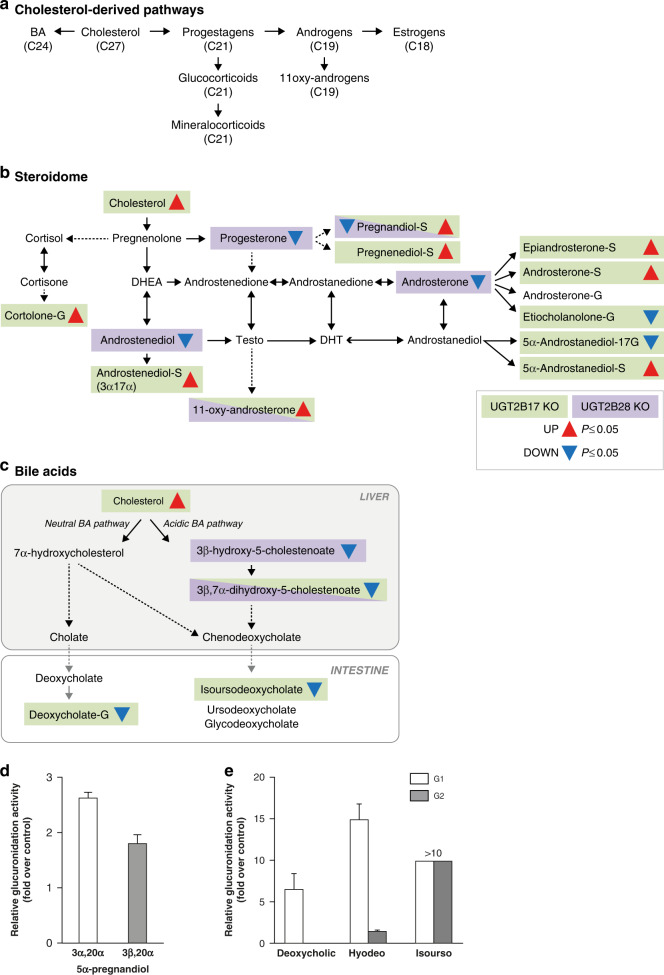
Steroidome and biliary acids. **a** Overview of cholesterol-derived steroid and bile acid metabolism. The number of carbon (C) is indicated. **b** Quantitative changes in steroid hormones assessed by untargeted and targeted assays in *UGT2B17* KO and *UGT2B28* KO individuals relative to gene-proficient controls. **c** Quantitative changes and metabolic pathway of significantly changed bile acids (BA). In the hepatic primary BA pathway, a classical/neutral and an alternative/acidic pathway, respectively, produce cholic acid and chenodeoxycholate that are conjugated to taurine and glycine to facilitate transport through blood toward the intestine where they are further metabolised by human intestinal enzymes and the microbiota before reabsorption for enterohepatic circulation. Cholate and deoxycholate were not measured. Detailed quantitative data are provided in Supplementary Table S3A (BA) and Supplementary Table S4A (steroids). Testo testosterone, DHT dihydrotestosterone, DHEA dehydroepiandrosterone, BA bile acid. **d** 5α-pregnandiol glucuronides are formed by LNCaP prostate cancer cells overexpressing UGT2B28; **e** Conjugation of the bile acids deoxycholic acid, hyodeoxycholic acid (hyodeo; also named 3α,6α-dihydroxy-5β-cholan-24-oic acid) and isoursodeoxycholic acid (isourso) by LAPC4 prostate cancer cells overexpressing UGT2B17. Glucuronidation activity is represented relative to cells transfected with an empty vector. Chromatograms and fragmentation profiles of glucuronidated derivatives are shown in Supplementary Fig. S2A–D.

We examined in depth the impact of each complete gene deletion on circulating levels of steroid hormones, known substrates of UGT2B17 and UGT2B28 enzymes. Cholesterol is the 27-carbon metabolic precursor of both steroid hormones and bile acids (Fig. 3a), two classes of globally perturbed metabolites in KO cases. Cholesterol was significantly higher in *UGT2B17* KO (FC 1.14X, *P* < 0.01) but unchanged in *UGT2B28* KO relative to gene-proficient individuals (Fig. 3b). The untargeted metabolomics analysis measured 35 steroids (mainly steroid sulfate and glucuronide conjugates) whereas two targeted and complementary steroid analyses measured 22 steroids including few sulfate and glucuronide conjugates (Supplementary Tables S3A and S4A).

The steroidome of *UGT2B17* KO individuals was largely affected, with higher levels of seven sulfate conjugates (FC 1.3–2.1X), lower levels of three glucuronide conjugates (etiocholanolone-G, FC −1.5X; 5α-androstan-3α,17β-diol-17-G (3α-diol-17-G), FC −1.7X; *P* < 0.05), higher levels of cortolone-G (1.2X, *P* < 0.05), and higher levels of 11-oxy-androsterone (1.6X, *P* < 0.05) (Fig. 3b and Supplementary Tables S3A and S4A). Androsterone and 3α-diol are known substrates of the UGT2B17 enzyme [44]. The impact of *UGT2B28* KO on the circulating steroidome was also significant but divergent. No changes in glucuronide steroid derivatives but a lower level of the C19 derivative androstenediol (FC −1.4X, *P* < 0.05), and higher 11-oxy-androsterone (FC 1.9X, *P* < 0.05) were observed in *UGT2B28* KO (Fig. 3B). *UGT2B28* KO was also associated with reduced 5α-pregnan-3β,20β-diol sulfate (FC −1.3X, *P* < 0.05). Functional glucuronidation assays support that the pregnanediol derivatives 5α-pregnan-3α,20α-diol and 5α-pregnan-3β,20α-diol are conjugated by UGT2B28 in LNCaP prostate cancer cells (Fig. 3d and Supplementary Fig. S1A). For C18 oestrogens, no significant changes in the levels of three oestrogenic metabolites measured in plasma were observed in UGT KO cases. However, using data from a previous targeted analysis of urine samples from a subset of PCa cases studied herein, we observed that *UGT2B28* KO displayed drastically lower urine levels of 2-methoxy estradiol by (FC 4.2X, *P* < 0.05) (Supplementary Table S4A).

Also derived from cholesterol, the alternative/acidic bile acid pathway was similarly affected in both KO groups, with significantly lower cholesterol-derived 3β-hydroxy- and 3β, 7α-dihydroxycholestenoate (−1.2X to −1.4X, *P* < 0.01) whereas metabolites of the neutral pathway were unchanged (Fig. 3c and Supplementary Table S3A). The chenodeoxycholate-derived secondary metabolite isoursodeoxycholate was lower by −2.3X (*P* < 0.05) in *UGT2B17* KO). *UGT2B17* KO individuals also had remarkably lower levels of deoxycholate-glucuronide by −8.3X (*P* < 10^–6^), which constituted a circulating metabolite discriminating *UGT2B17* KO from proficient individuals (Fig. 3c). In support, several bile acids including deoxycholic, hyodeoxycholic and isoursodeoxycholic acids, were efficiently conjugated in LAPC4 prostate cancer cells overexpression UGT2B17 (Fig. 3e and Supplementary Fig. S1B–D).

### The circulating lipidome is largely perturbed in *UGT* KO individuals

The levels of multiple circulating lipid species diverged between KO groups and with gene-proficient PCa cases (Fig. 2c, e).

#### Fatty acid and acylglycerol metabolites

*UGT2B17* KO displayed a distinctive global enrichment of circulating triacyglycerol carrying docosahexanoic acid (TAG_DHA; Fig. 2e) whereas *UGT2B28* KO cases were characterised by globally reduced carnitine (−13%, *P* = 0.007), short and medium chain fatty acid acylcarnitines (−22% to −32%, *P* ≤ 0.05) and fatty acid dicarboxylates (−22% to −45%; *P* ≤ 0.05) (Fig. 2d). None of the free fatty acid species (short, medium or long chain, saturated, monounsaturated (MUFA), polyunsaturated (PUFA) or branched), including DHA and its precursor α-linolenic acid, were significantly affected (Supplementary Table S3A).

#### Sphingolipids

**Fig. 4:**
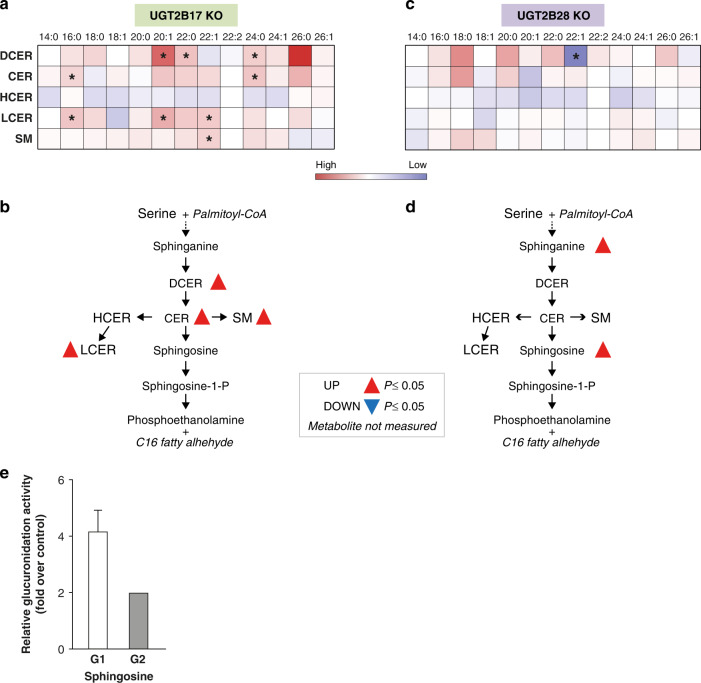
*UGT2B17* KO and *UGT2B28* KO distinctively perturb the sphingolipid pathway. **a** Relative quantitative changes in sphingolipid levels in *UGT2B17* KO vs gene-proficient controls. *P ≤ 0.05. **b** Changed sphingolipids in UGT2B17 KO are highlighted in the metabolic pathway; *P ≤ 0.05. **c** Relative quantitative changes in sphingolipids levels in *UGT2B28* KO vs gene-proficient controls. **d** Changed sphingolipids in UGT2B28 KO are highlighted in the metabolic pathway. Metabolites not measured are italicised in pathways. Detailed quantitative data are provided in Supplementary Table S3. CER ceramides, DCER dihydroceramides, HCER hexosylceramides, LCER lactosylceramides, SM sphingomyelins. **e** Two glucuronidated sphingosine conjugates (G1 and G2) are formed by LAPC4 prostate cancer cells overexpressing UGT2B28. Glucuronidation activity is represented relative to cells transfected with an empty vector. Chromatogram and fragmentation profiles of glucuronidated sphingosine derivatives are shown in Supplementary Fig. S2E.

Several classes of sphingolipids were significantly upregulated only in *UGT2B17* KO. This included levels of dihydroceramides, ceramides, lactosylceramides and sphingomyelins, increased by 10–38% in *UGT2B17* KO (Figs. 2d and 4a, b). By contrast, *UGT2B28* KO were characterised by higher levels of the ceramide precursors sphinganine (51%, *P* ≤ 0.05) and sphingosine (45%, *P* = 0.02) (Figs. 2d and 4c, d). This observation is supported by the detection of two glucuronide derivatives of sphingosine by mass spectrometry and their increased formation in the UGT2B17-deficient prostate cancer LAPC4 cells expressing UGT2B28 over control cells (Fig. 4e and Supplementary Fig. S1E).

#### Eicosanoids/oxylipins

**Fig. 5:**
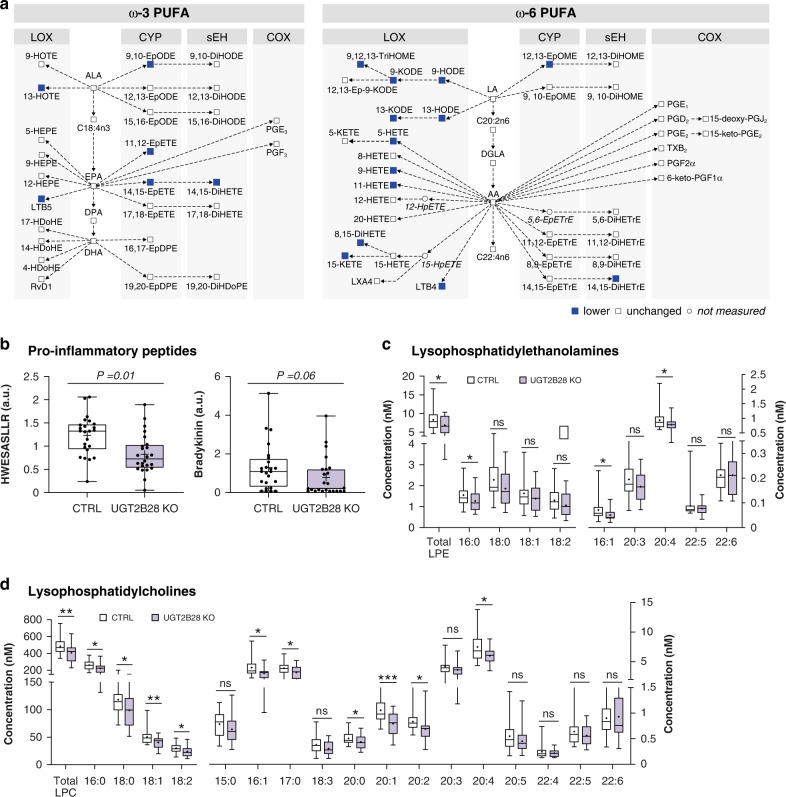
*UGT2B28* KO display lower inflammation mediators in circulation. **a** LOX and CYP/sEH-derived oxylipins are lower in *UGT2B28* KO than gene-proficient controls. COX-derived oxylipins are not changed relative to control cases blue squares: lower oxylipin; full name and quantitative metabolomics data are provided in Supplementary Table S4B. **b** The pro-inflammatory peptides HWESASLLR and the bradykinin, derived from the kallikrein–kinin system, are lower in UGT2B28 KO; Levels of **c** lysophosphatidylethanolamine and **d** lysophosphatidylcholine in control and UGT2B28 KO cases. The median (line) and mean (+) values are indicated in boxplots. *P ≤ 0.05; **P ≤ 0.01; ***P ≤ 0.001. ns not significant, LOX lipoxygenase, COX cyclooxygenase, CYP cytochrome P450, sEH soluble epoxyhydrogenase, AA arachidonic acid, ALA α-linolenic acid, DGLA dihomo-γ-linolic acid, DHA docosahexanoic acid, DPA doocosapentanoic acid, EPA eicosapentanoic acid, LA linoleic acid, LPC lysophosphatidylcholine, LPE lysophosphatidylethanolamine. Quantitative metabolomics data for B, C and D are provided in Supplementary Table S3A.

There was a remarkable global perturbation of eicosanoids in *UGT2B28* KO individuals, first hinted by the untargeted metabolomics assay (Fig. 2d). An exhaustive targeted analysis of oxylipins further revealed lower levels of 11 of 69 quantified eicosanoids (Fig. 5a and Supplementary Table S4B). Oxylipins derived from the lipoxygenase (LOX)-dependent oxygenation of ω-6 PUFA were the most perturbed, with 8 of the 17 measured (47%) that were reduced by 17–72% relative to UGT-proficient controls (Fig. 5a). Several cytochrome P450 and soluble epoxygenase-derived oxylipins were also lower in *UGT2B28* KO individuals whereas the COX-derived prostaglandins (prostanoids) were unaffected. These observations were paralleled by lower systemic levels of other inflammatory mediators in *UGT2B28* KO, namely of the pro-inflammatory peptides bradykinin and HWESASLLR, associated with the kallikrein–kinin system [45, 46] and of lysophosphatidylcholine (LPC) and lysophosphatidylethanolamine (LPE) levels (Figs. 2d, e and 5b, c). LPCs were broadly lower by 14–25% for 10 out of 17 measured species (Fig. 5c), and 3 out of 9 measured LPEs, from which LPCs may be produced, were also globally lower by 16–29% (Fig. 2e). Glycerophosphorylcholine (GPC) and glycerophosphoethanolamine (GPE) were also significantly reduced by 14% and 17%, respectively, in *UGT2B28* KO cases (Fig. 2d), whereas very few phosphatidylcholines and phosphatidylethanolamines were perturbed. None of these metabolites were significantly changed in *UGT2B17* KO versus control cases.

### *UGT2B17* and *UGT2B28* KO inversely impact the tryptophan/kynurenine pathway

Amino acids were another class of metabolites broadly affected in both *UGT2B17* and *UGT2B28* KO (Fig. 2c). *UGT2B17* KO were characterised by changes in the arginine and lysine pathways and higher levels (by 16–45%) of several gamma-glutamyl amino acids whereas *UGT2B28* KO displayed a reduced branched-chain amino acid metabolism (Fig. 2d and Supplementary Table S3A). The tryptophan/kynurenine pathway was also differently perturbed in KO groups (Supplementary Fig. S2). *UGT2B17* KO were characterised by significantly higher levels of kynurenine by 21% relative to gene-proficient controls. In vitro glucuronidation assays do not support conjugation of tryptophan and kynurenine with glucuronic acid (data not shown), whereas *UGT2B28* KO had unchanged kynurenine levels, but reduced levels of anthranilate, by 39% (Supplementary Table S3A).

### Carbohydrates, energy and nucleotide pathways

Glycosyltransferases use UDP-glucuronic acid (UDP-GlcA) as a sugar donor in the conjugation reaction. We thus examined metabolites linked to the uronic and hexosamine pathways, which are synthesised from glycolytic intermediates. In *UGT2B17* KO cases, none of the measured glycolytic intermediates and metabolites of the tricarboxylic acid pathway were perturbed (Supplementary Table S3A and Supplementary Fig. S2B). *UGT2B28* KO displayed significantly higher levels of the glycolytic metabolite pyruvate by 53% and of N-acetylglucosamine/galactosamine of the hexosamine pathway by 29% (Supplementary Fig. S2B). Circulating nucleotide levels were globally unchanged between *UGT* KO and gene-proficient individuals (Supplementary Table S3A).

## Discussion

Our study highlights the potential of exploiting naturally occurring human gene KO to interrogate global gene functions and identify metabolic changes caused by gene loss. Our observations demonstrate that *UGT2B17* and *UGT2B28* complete gene deletions differentially influence the systemic metabolome, affecting levels of important classes of metabolites. Some of these metabolites were reported to be altered in previous PCa metabolomic studies and to promote PCa cancer growth and invasion/metastasis, such as steroids and ceramides [24, 47–49]. This is consistent with the observations that gene copy number (CNV) and *UGT2B17* and *UGT2B28* expression levels have been associated with the progression of PCa [10, 13, 14, 17, 20]. A key observation is the different metabolomic perturbations in the circulation of *UGT2B17* and *UGT2B28* KO PCa cases, despite a shared 84.3% nucleotide sequence identity between the two genes, a similar tissue distribution (present in hepatic and prostate tissues) and overlapping steroidogenic substrates (Fig. 1 and Supplementary Fig. S1) [25, 44]. It was unanticipated to observe divergent metabolic perturbations influencing same metabolic pathways, including the steroidome and ceramide/sphingolipid pathways. The distinctive metabolites affected in each pathway support that UGT2B17 and UGT2B28 have different key functional roles and reinforce the need to examine the unique functions of UGT proteins in PCa biology.

*UGT2B17* KO individuals were distinguished by reduced levels of steroid-glucuronide conjugates and higher sulfated derivatives, a discriminatingly lower level of the bile acid deoxycholate-glucuronide, and higher levels of several ceramides. *UGT2B28* KO individuals were characterised by an overall lower level of steroids, fatty acid carnitines and dicarboxylates, as well as oxylipins and other inflammatory mediators. Because of their known glycosyltransferase activity towards endogenous substrates (Fig. 1), one may expect that at least some changes are directly attributable to a reduced ability to conjugate these metabolites. This is supported by functional assays with various metabolites, such as deoxycholate and sphinganine, for which levels were significantly affected in *UGT2B17* KO and *UGT2B28* KO individuals, respectively. However, several changes observed are not explained by known substrates of the UGT2B17 or UGT2B28 enzymes, such as kynurenine, or do not belong to classes of metabolites previously demonstrated to be conjugated by UGT enzymes, suggesting additional functions.

Glycosyltransferases, including UGT2B17 and UGT2B28, are appreciated as key regulators of the bioavailability of sex steroid hormones and action, likely explaining their links with several hormone-sensitive diseases, including PCa [24]. Our current study supported the key role of UGT2B17 in the inactivation of the DHT metabolite androstane-3α,17β-diol-17G and also highlighted a connection of UGT2B17 and UGT2B28 to adrenal steroid precursors, progestins and cortisone derivatives. These observations are in keeping with our previous findings conducted on smaller cohorts of PCa patients that compared the influence of variations in *UGT2B17* and *UGT2B28* CNV and with the known conjugation substrate preference of these UGTs [10, 13, 25, 44]. However, no other studies have profiled the steroidome of *UGT2B28* KO individuals, for which the enzymatic function has been explored in a single previous study [25]. Our observations are also consistent with studies conducted in serum and urine, reporting an influence of the *UGT2B17* gene status on the steroidome [27, 50]. A phenomenon observed in *UGT2B17* KO individuals was the adaptive compensatory steroid sulfation pathway that paralleled the reduced levels of glucuronide conjugation, suggesting an intimate cross-talk between these two conjugation pathways for the regulation of steroids bioavailability. The increased urinary level of etiocholanolone-sulfate in healthy male individuals with *UGT2B17* gene loss is consistent with this notion [28]. Steroids are metabolites where both sulfation and glucuronidation represent effective pathways to increase the hydrophilic nature of the steroids, but with different biological consequences. Glucuronides are considered end products for elimination, whereas sulfates may contribute to the pool of precursor or bioactive steroids [37, 51]. The production of sulfate over glucuronide conjugates may thus contribute to PCa progression and involve a different regulation of signalling pathways by sex steroids.

With the comprehensive metabolic profiling presented here, we identified a broad systemic metabolic rewiring of lipid pathways such as ceramides/sphingolipids. *UGT2B17* KO presented increased levels of ceramide species, whereas it was rather the ceramide precursors sphinganine and sphingosine that were elevated in *UGT2B28* KO relative to gene-proficient cases. It is intriguing that in *UGT2B28* KO, largely defined by a global reduction of most measured metabolites, sphingolipid-related metabolites were higher than in controls, suggesting an accumulation caused by a defect in their conjugation by UGT2B28. This postulate is supported by functional assays demonstrating a significant accumulation of sphingosine-glucuronide in LNCaP PCa cells expressing UGT2B28 (Fig. 4e and Supplementary Fig. S1E). An endocrine regulation of lipid metabolism, including fatty acids, ceramides and other sphingolipids, has been recently uncovered in PCa. This androgen receptor-lipid axis was associated with cancer progression and drug response and is considered as a therapeutic vulnerability [47, 48, 52, 53]. In addition, circulating ceramide levels were recently associated with poor clinical outcomes across localised and metastatic castration-sensitive and castration-resistant PCa [48], consistent with a more aggressive disease in germline *UGT2B17* and *UGT2B28* KO than gene-proficient cases [10, 20]. This is supported by their capacity to promote PCa growth [49].

Circulating levels of bile acids were reduced in both *UGT* KO groups, consistent with the expression of UGT2B17 and UGT2B28 in the liver and gastrointestinal tract (Supplementary Fig. S1). A functional assay demonstrating that deoxycholate is a substrate of the UGT2B17 enzyme suggests a direct modulatory effect of UGT2B17 on the levels of deoxycholate-glucuronide, a metabolite discriminating *UGT2B17* KO from proficient individuals. Consistent with our observation, deoxycholate was shown to be elevated in men with metastatic PCa in a prospective metabolomics study [54]. Furthermore, this specific secondary bile acid lipid was involved in promoting cancer growth and invasion/metastasis, namely through the regulation of β-catenin signalling [55]. The lower levels of LPC 16:0 and 18:0 observed in *UGT2B28* KO is consistent with previous observations linking lower levels of LPC (16:0) to biochemical recurrence of PCa after prostatectomy and lower plasma levels of LPC (18:0) to an increased risk of PCa [56, 57]. Amongst LPC functions, they act as pro-inflammatory lipids involved in the pathogenesis of inflammatory diseases [58]. The overall lower circulating lipid mediators of inflammation, namely LOX- and CYP-derived oxylipins, LPCs and inflammatory peptides were thus another intriguing hallmark of *UGT2B28* KO individuals. Because the inflammatory shift affected many oxylipins and other inflammatory metabolites, a feedforward loop or mechanism independent of the known enzymatic activity of UGT2B28, such as protein interactions between UGT2B28 and biosynthetic enzymes such as LOX or CYPs, may be envisioned. Consistent with this hypothesis, functional protein interactions between other UGTs and CYP enzymes have been reported, with an influence on enzymatic functions and cancer cell phenotypes [59, 60]. Besides, little is known about the inflammatory peptide HWESASLLR associated with *UGT2B28* KO, which appears related to levels of bradykinin [45], also reduced in *UGT2B28* KO. Both peptides are produced by kallikrein peptidases that include the PSA encoded by KLK3, used in the screening and monitoring of PCa [46].

In *UGT2B17* KO, the higher kynurenine and downstream metabolites suggested activation of this pathway. Higher kynurenine concentrations have been reported in prostatic tumoral tissues and in serum samples of PCa cases compared to controls and are associated with a more aggressive disease [61–64] in keeping with an increased risk of progression for PCa individuals with germline *UGT2B17* KO. However, the mechanism by which UGT2B17 affects the kynurenine pathway remains to be elucidated and does not seem to be caused by direct glucuronidation of tryptophan or kynurenine on the basis of our functional in vitro assays (not shown).

Limitations of this study focused on PCa include that only men were analysed and the consequences of *UGT* KO remain to be fully examined. As blood samples were collected at surgery prior to removal of primary tumours, metabolic profiles may reflect in part tumour activity in addition to the systemic disease state in the context of *UGT* KO. Nonetheless, control PCa cases were carefully matched for the aggressiveness of the disease as well as patient age, limiting the impact of disease-specific metabolic changes. UDP-activated sugars, including the co-substrate UDP-GlcA preferentially used in the glucuronidation reaction by UGT2B enzymes, were not measured by the MS approaches used. The study confirms our hypothesis of a broad metabolic rewiring caused by germline UGT KOs. The circulating metabolome reflects the overall impact of the complete absence of UGT2B17 and UGT2B28 in each tissue where they are normally expressed. The metabolic activity of UGTs in each tissue, and also their interplay with other metabolic pathways, are likely to contribute to the systemic metabolic alterations associated with UGT KO. Some of the observed changes may represent the consequences of direct conjugation of metabolites but also adaptive metabolism in the absence of UGT enzyme, indirectly resulting from the perturbed homoeostasis of endogenous metabolites created by the *UGT* gene losses and feedforward regulatory loops. Other mechanisms may be involved, such as protein–protein interactions with an impact on metabolite levels possibly unrelated to their transferase activity. Our studies have recently hinted that the UGT enzymes may participate in other cellular and metabolic functions by this process that is also supported by their subcellular localisation not limited to the endoplasmic reticulum and depending on their tissue-specific expression [24].

Given the relatively low frequency of the *UGT* KO genotypes, especially for *UGT2B28*, this study is the first to include sufficient cases to inform on global metabolic perturbations of these complete human gene KO. The important classes of metabolic pathways modified in *UGT2B17* KO are elevated ceramides, kynurenine and triacylglycerol, all associated with adverse PCa outcomes [47, 65, 66], whereas more broadly reduced metabolites are observed in *UGT2B28* KO, including oxylipins and other inflammatory mediators but with an increase in ceramides/sphingolipids precursors, also linked to PCa cancer growth and invasion/metastasis [48, 49]. Our study thus delineates divergent metabolomes by individual *UGT* gene loss at the systemic level, well beyond steroidogenesis, providing potential novel insights on how UGT2B17 and UGT2B28 may differentially influence the course of PCa. Findings may also be relevant to several other clinical conditions also associated with UGT KO, including solid and haematological cancers, bone mineral density and osteoporosis, and autoimmune diseases [11, 12, 16, 18, 19, 22, 30, 67, 68]. Metabolic changes linked to *UGT* KO pinpoint possible vulnerabilities that deserve further characterisation, including those of the steroidome and inflammation.

## Supplementary information

Supplemental Material

Supplementary Table S3A

Supplementary Table S3B

Supplementary Table S4A

Supplementary Table S4B

Supplementary Table S5

Reporting Summary form

## Data Availability

Not applicable.
