# Supplementary material for: Extensive metabolic consequences of human glycosyltransferase gene knockouts in prostate cancer
Source: Br J Cancer. 2022 Nov 8;128(2):285–96. doi: 10.1038/s41416-022-02040-w (PMC9902621; doi:10.1038/s41416-022-02040-w)
Supplement: Supplementary file 1 — Supplemental Material [file 41416_2022_2040_MOESM1_ESM.pdf]

Extensive metabolic consequences of human glycosyltransferase gene knockouts in prostate cancer

Michèle Rouleau[1], Flora Nguyen Van Long[1], Véronique Turcotte[1], Patrick Caron[1], Louis Lacombe[2], Armen Aprikian[3], Fred Saad[4], Michel Carmel[5], Simone Chevalier[3], Eric Lévesque[2] and Chantal Guillemette[1†]

[1]Centre Hospitalier Universitaire de Québec Research Center - Université Laval (CRCHUQc-UL), Faculty of Pharmacy and Centre de Recherche sur le Cancer, Université Laval, Québec, Canada

[2]CRCUQc-UL and Faculty of Medicine - Université Laval, Québec, Canada

[3]McGill University Health Centre, McGill University, Faculty of Medicine, Québec, Canada.

[4]Centre Hospitalier de l'Université de Montréal, Faculty of Medicine, Université de Montréal, Québec, Canada.

[5]Université de Sherbrooke, Faculty of Medicine, Québec, Canada.

Supplementary Information:

Supplementary Table S1: Overview of metabolomics data

Supplementary Table S2: Most changed metabolites in UGT2B17 KO and UGT2B28 KO

Supplementary Table S3 (Excel spreadsheets): Detailed global metabolomics and lipidomics quantitative data

Supplementary Table S4 (Excel spreadsheets): Detailed steroid and oxylipin quantitative data

Supplementary Table S5 (Excel spreadsheet): Metabolomics quantitative data per patient

Figure S1: Tissue expression profiles of UGT2B17 and UGT2B28.

Figure S2: Novel UGT2B17 and UGT2B28 substrates: Chromatograms and fragmentation profiles of glucuronide derivatives by mass spectrometry analysis (MS).

Figure S3: Metabolic perturbations in the tryptophan-kynurenine and glucose-derived pathways in *UGT2B17* KO and *UGT2B28* KO

Table S1 – Overview of metabolomics data

MS-based analysis	UGT2B17 KO						UGT2B28 KO					
	metabolites changed *vs* gene proficient cases			n (% of changed)[6]			metabolites changed *vs* gene proficient cases			n (% of changed)[6]		
n=measured metabolites[1]		n	%[5]	up	down			n	%	up	down	
**Global (untargeted)**[2] n=651	**P≤0.05**	**48**	**7.4**	**26 (54%)**	**22 (46%)**		**P≤0.05**	**56**	**8.6**	**11 (20%)**	**45 (80%)**	
	P<0.1	39	6	14 (36%)	25 (64%)		P<0.1	47	7.2	9 (19%)	38 (81%)	
**Lipidomics**[2] n=804	**P≤0.05**	**37**	**4.6**	**32 (86%)**	**5 (14%)**		**P≤0.05**	**19**	**2.4**	**1 (5%)**	**18 (95%)**	
	P<0.1	49	6	36 (73%)	13 (17%)		P<0.1	17	2.1	4 (24%)	13 (76%)	
**Oxylipins**[3] n=68	**P≤0.05**	**2**	**2.9**	**0**	**2 (100%)**		**P≤0.05**	**11**	**16.2**	**0**	**11 (100%)**	
	**P<0.1**	**1**	**1.5**	**0**	**1 (100%)**		**P<0.1**	**5**	**7.3**	**0**	**5 (100%)**	
**Steroids** (assay #1)[4] n=15	**P≤0.05**	**1**	**6.7**	**0**	**1 (100%)**		**P≤0.05**	**1**	**6.7**	**0**	**1 (100%)**	
	P<0.1	0	0	0	0		P<0.1	2	13.3	0	2 (100%)	
**Steroids** (assay #2)[4] n=7	**P≤0.05**	**1**	**16.7**	**1 (100%)**	**0**		**P≤0.05**	**1**	**16.7**	**1 (100%)**	**0**	
	P<0.1	0	0	0	0		P<0.1	0	0	0	0	
**Total** n=1545	**P≤0.05**	**89**	**5.8**	**59 (66%)**	**30 (34%)**		**P≤0.05**	**88**	**5.7**	**13 (15%)**	**75 (85%)**	
	P<0.1	89	5.8	50	39		P<0.1	71	4.6	13	58	

[1]Measured in plasma from male prostate cancer patients (matched for characteristics presented in Table 1).
[2]Untargeted global metabolomics and lipidomics were performed with the Metabolon plateform.
[3]Oxylipins were measured by the West Coast Metabolomics Center.
[4]Steroids were measured in-house using two multiplex assays measuring adrenal precursors, androgens and estrogens (assay #1) and 11-oxygenated steroids (assay #2).
[5]% of measured metabolites.
[6]% of changed metabolites.

Table S2 – Most changed metabolites in *UGT2B17* KO and *UGT2B28* KO

UGT2B17 KO		UGT2B28 KO	
5α-androstan-3β,17α-diol disulfate	2.07	**adipoylcarnitine**	2.70
adipate	1.69	glycerol	1.91
androsterone sulfate	1.61	sphinganine	1.55
androstenediol (3α,17α) monosulfate	1.61	2-keto-3-deoxy-gluconate	1.55
nervonoylcarnitine (C24:1)	1.50	pyruvate	1.53
5α-androstan-3α,17β-diol monosulfate	1.49	mannonate	1.52
hydroxy-CMPF[1]	1.46	sphingosine	1.47
gamma-glutamyl-2-aminobutyrate	1.45	succinoyltaurine	1.31
epiandrosterone sulfate	1.41	5-(galactosylhydroxy)-L-lysine	1.30
N-acetylcarnosine	1.39	N-acetylglucosamine/N-acetylgalactosamine	1.29
**deoxycholic acid glucuronide**	-8.33	eicosenedioate	-1.82
trigonelline (N'-methylnicotinate)	-2.33	1-oleoyl-GPG (18:1)[2]	-1.72
isoursodeoxycholate	-2.33	N,N-dimethyl-5-aminovalerate	-1.61
2,3-dihydroxyisovalerate	-2.22	N6-methyllysine	-1.59
5-acetylamino-6-amino-3-methyluracil	-1.96	N,N,N-trimethyl-5-aminovalerate	-1.59
pipecolate	-1.89	isobutyrylcarnitine	-1.49
N2-acetyl,N6,N6-dimethyllysine	-1.82	HWESASLLR	-1.49
homocitrulline	-1.61	propionylcarnitine	-1.47
cysteinylglycine disulfide	-1.56	3β,17α-dihydroxy-5-cholestenoate	-1.45
5α-androstan-3α,17β-diol 17-glucuronide	-1.56	glutarate	-1.45
		laurylcarnitine	-1.45

[1]CMPF: 3-carboxy-4-methyl-5-propyl-2-furanpropanoate
[2]GPG: glycerophosphoglycerol

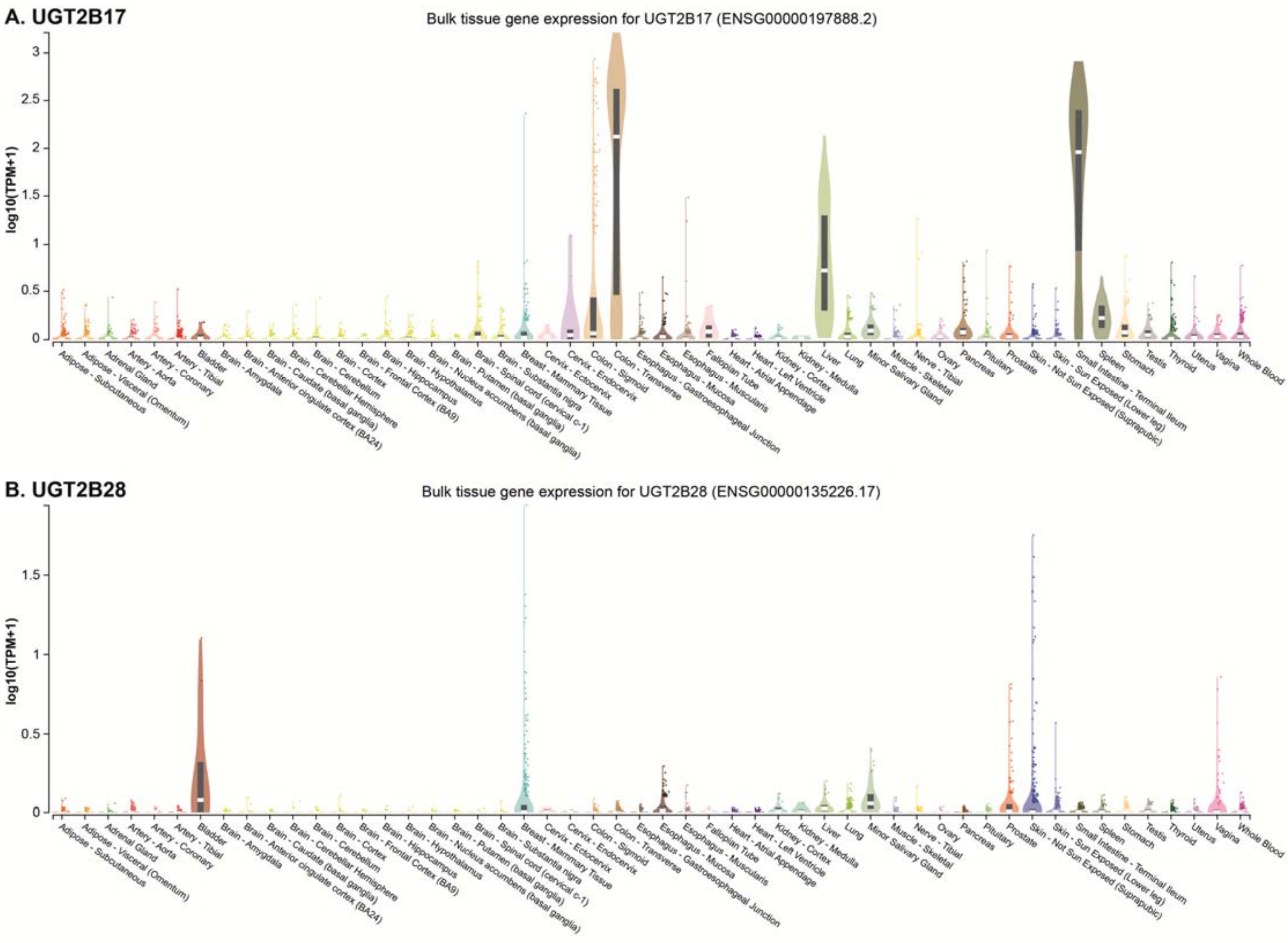

Fig.S1. Tissue expression profiles of UGT2B17 and UGT2B28. Gene expression levels were reported according to the GTEx portal (accessed on June 20, 2022; https://gtexportal.org/home/)

TPM: transcripts per million read.

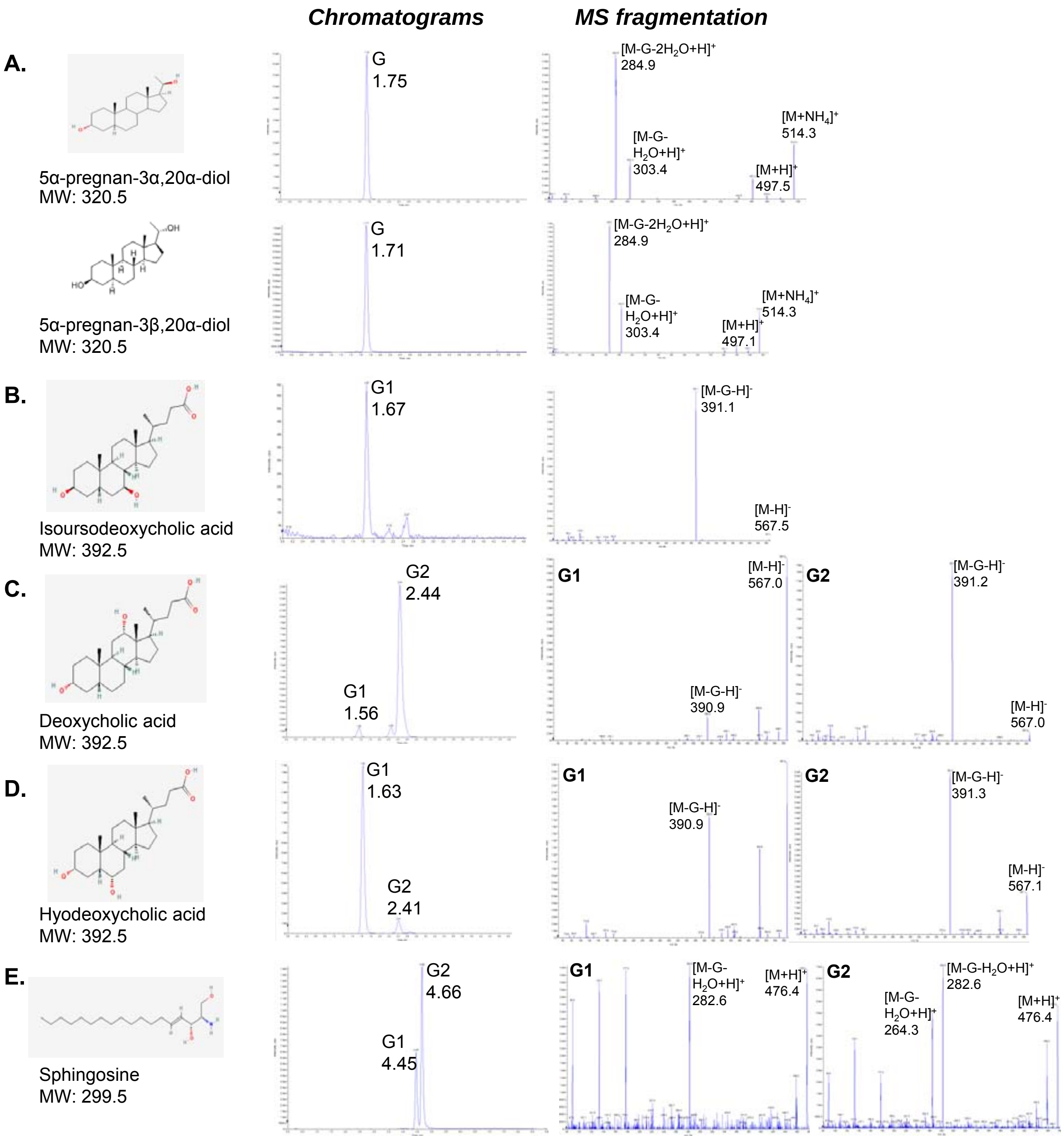

Fig.S2. Novel UGT2B17 and UGT2B28 substrates: Chromatograms and fragmentation profiles of glucuronide derivatives by mass spectrometry analysis (MS). MS analysis indicated the formation of one or two glucuronidated products named G1 and G2 according to their order of elution. **A.** 5α-pregnandiols glucuronide (G) conjugation by LNCaP prostate cancer cells overexpressing UGT2B28; B-D. Glucuronide of bile acids formed by LAPC4 prostate cancer cells overexpressing UGT2B17. E. Sphingosine glucuronide derivatives formed by LAPC4 prostate cancer cells overexpressing UGT2B28. Glucuronides were also formed by microsomes of human liver, expressing both UGT2B17 and UGT2B28. Chromatograms indicate elution time of each glucuronide. Fragmentation patterns assessed by MS show the loss of the GlcA moiety (M-G) corresponding to a m/z shift of 176 Da relative to the glucuronidated compound (M+H).

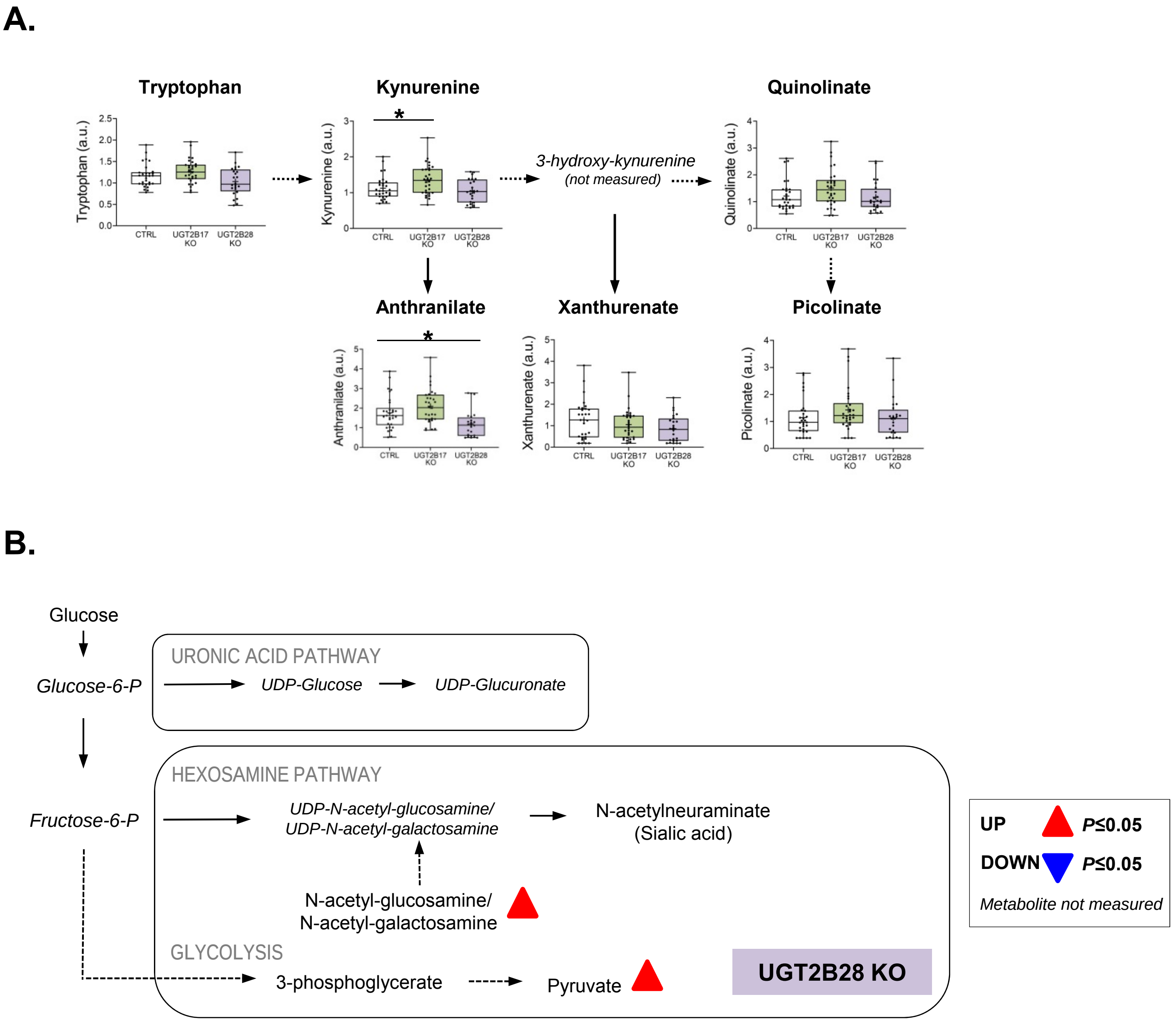

Fig.S3. Distinctive changes in the tryptophan-kynurenine and glucose-derived pathways in *UGT2B17* KO and *UGT2B28* KO. A. Kynurenine is elevated in *UGT2B17* KO individuals. Kynurenine and several other tryptophan-derived metabolites are higher than controls in *UGT2B17* KO. In *UGT2B28* KO, fewer metabolites are changed, with only anthranilate significantly reduced relative to controls. Boxes represent the interquartile range (25-75%), whiskers indicate maximum and minimum values, the median and mean values are indicated by a solid line and a + sign, respectively. Significant changes are indicated. Tryptophan-derived metabolites that were unchanged are not shown. **B.** Metabolic perturbations of the glucose-derived uronic acid and hexosamine pathways in *UGT2B17* KO and *UGT2B28* KO cases. Quantitative data are provided in Table S3A.
